# Complete Genome Sequence of Vitreoscilla sp. Strain C1, Source of the First Bacterial Hemoglobin

**DOI:** 10.1128/MRA.00922-18

**Published:** 2018-08-09

**Authors:** Iva A. Veseli, Anne Caroline Mascarenhas dos Santos, Oscar Juárez, Benjamin C. Stark, Jean-François Pombert

**Affiliations:** a Department of Biology, Illinois Institute of Technology, Chicago, Illinois, USA; Louisiana State University

## Abstract

Vitreoscilla sp. strain C1 is of historical importance as the source of the first prokaryotic hemoglobin identified. Vitreoscilla spp. rely on their hemoglobin and cytochrome oxidase to grow in microaerobic environments despite their aerobic nature. To help characterize this historically relevant strain, we sequenced the complete Vitreoscilla sp. strain C1 genome.

## Abstract (precis)

Vitreoscilla sp. strain C1 is of historical importance as the source of the first prokaryotic hemoglobin identified.

## ANNOUNCEMENT

Vitreoscilla spp. are obligate aerobic proteobacteria known for their remarkable respiratory adaptations, which enable them to grow under hypoxic conditions (1). Investigation of the Vitreoscilla sp. strain C1 metabolism culminated in the identification of the first prokaryotic hemoglobin, Vitreoscilla hemoglobin (VHb), and of its cytochrome oxidase (cytochrome *bo*), which pumps Na^+^ ions instead of H^+^ as reported in many other bacteria (e.g., Escherichia coli) (1–4). Despite the extensive characterization of Vitreoscilla sp. strain C1’s VHb and cytochrome *bo* and their biotechnological applications (5–8), the genotype of this historically important strain was never determined. To catalog this landmark strain and gain insights into its complete metabolic potential, we sequenced the complete genome of Vitreoscilla sp. strain C1.

Vitreoscilla sp. strain C1 was obtained from R. G. E. Murray of Western Ontario University in 1962 and has been cultured by D. A. Webster, P.-Y. Chi, and B. C. Stark at the Illinois Institute of Technology since 1967. The strain was inoculated in liquid medium (1.3% peptone, 1.3% yeast extract [pH 8.0]), incubated for 72 h under agitation (150 rpm) at room temperature, pelleted by centrifugation (13,300 × *g*, 2 min), and stored at −20°C. DNA was extracted from frozen pellets with the MasterPure Complete DNA purification kit (Epicentre, Madison, WI, USA) and quantified by fluorometry on Qubit 2.0 with a broad-range double-stranded DNA (dsDNA) assay kit (Invitrogen, Carlsbad, CA, USA), and its quality was assessed by electrophoresis. The DNA library was prepared from 22.5 µg of starting material with the DNA template prep kit 3.0 (Pacific Biosciences, Menlo Park, CA, USA) and sequenced with one single-molecule real-time (SMRT) cell (P5-C4 chemistry) on a PacBio RS II instrument (Pacific Biosciences) at the University of Michigan DNA Sequencing Core (Ann Arbor, MI, USA).

Sequencing reads (13,798 reads; *N*_50_, 15,295) were assembled with the Hierarchical Genome Assembly Process 3 (HGAP3) protocol (default settings) from SMRT Analysis 2.3.0 (9), and the resulting unitigs were merged into one using the *de novo* assembly method (default parameters) from Geneious R7 (10). The genome (2,610,419 bp; 44.2% G+C content; 43× coverage) was circularized by detecting the overlapping ends of the final unitig with BLASTN (11) homology searches and by trimming the redundant segment with extractseq from EMBOSS 6.4 (12). Base calling was validated with the RS_sequencing.1 protocol from SMRT Analysis 2.3.0. The genome (2,079 proteins, 21 rRNAs, 87 tRNAs) was annotated with Prokka 1.11 (13) using the GenBank compliant mode and RNAmmer as the rRNA predictor. Protein functions assigned with Prokka (E-value, ≤1e−30) were validated by comparisons with InterProScan5 (14) searches (default parameters) and BLASTP (11) queries (E-value, 1e−20; –culling_limit, 10) against NCBI’s Neisseriaceae reference data sets and UniProt/Swiss-Prot databases (15). Discrepancies were curated manually with Artemis 16.0.0 (16) based on the sum of evidence presented by the individual predictors. The previously determined sequences of the VHb (17) and cytochrome *bo* loci (18) were confirmed.

### Data availability.

The Vitreoscilla sp. strain C1 genome was deposited in GenBank under the accession number CP019644.
